# Supplementary material for: Cryptococcus extracellular vesicles properties and their use as vaccine platforms
Source: J Extracell Vesicles. 2021 Aug 2;10(10):e12129. doi: 10.1002/jev2.12129 (PMC8329992; doi:10.1002/jev2.12129)
Supplement: Supplementary file 1 — Supporting Information [file JEV2-10-e12129-s008.pdf]

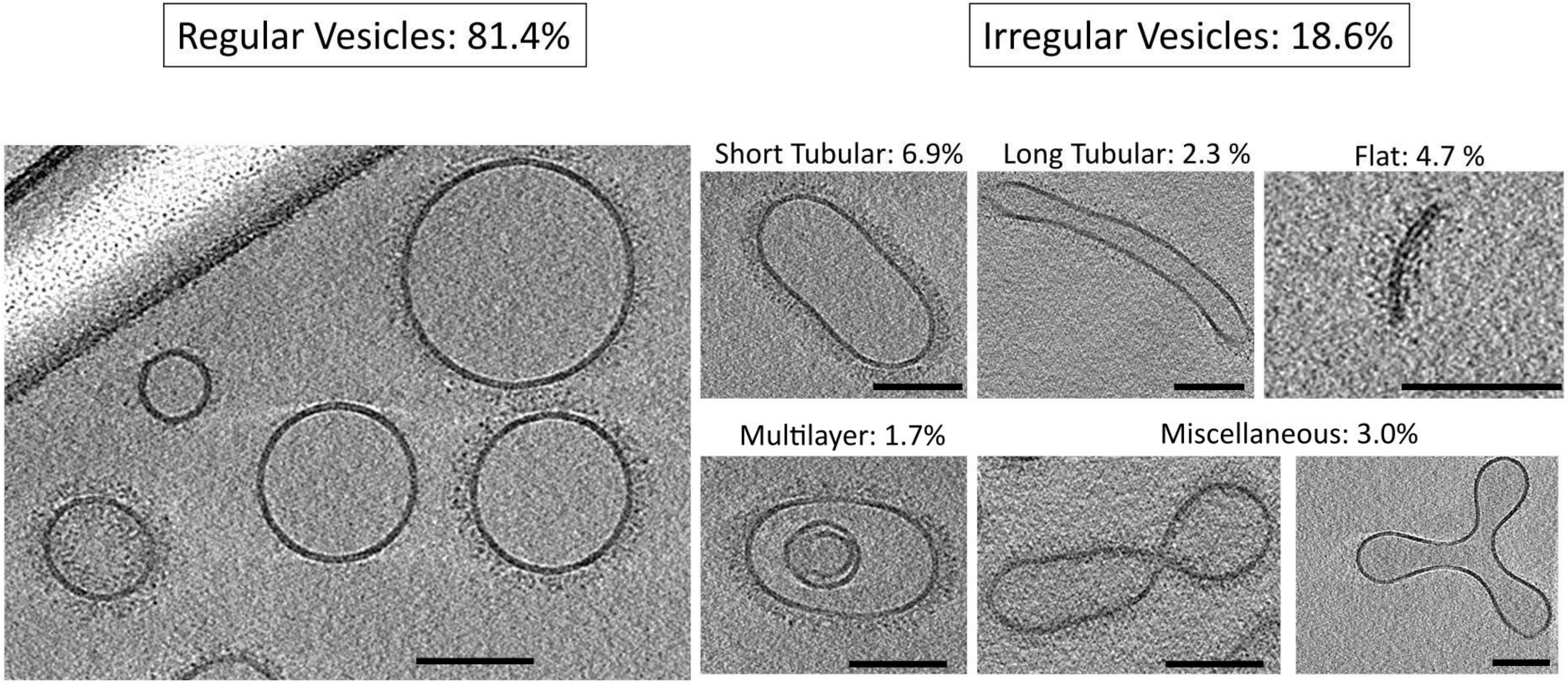

Figure S1: Gallery of EV categories. Cryo-EM analysis of 533 single EVs obtained from *C. neoformans*. EVs were characterized according to their morphological aspects in regular (rounded- bilayer vesicles) and irregular (not rounded – bilayer or multilayered vesicles) categories. Regular vesicles represented 81.4% of all EV analyzed. The irregular vesicles were subclassified as short tubular (6.9%), long tubular (2.3%), flat (4.7%), multilayer (1.7%), or vesicles with miscellaneous morphologies (3.0%). Scale bars represent 100nm. Data presented in this figure have been generated using images obtained using a Titan Krios (Thermo Scientific) transmission electron microscope.
