# Supplementary material for: Cryptococcus extracellular vesicles properties and their use as vaccine platforms
Source: J Extracell Vesicles. 2021 Aug 2;10(10):e12129. doi: 10.1002/jev2.12129 (PMC8329992; doi:10.1002/jev2.12129)
Supplement: Supplementary file 2 — Supporting Information [file JEV2-10-e12129-s005.pdf]

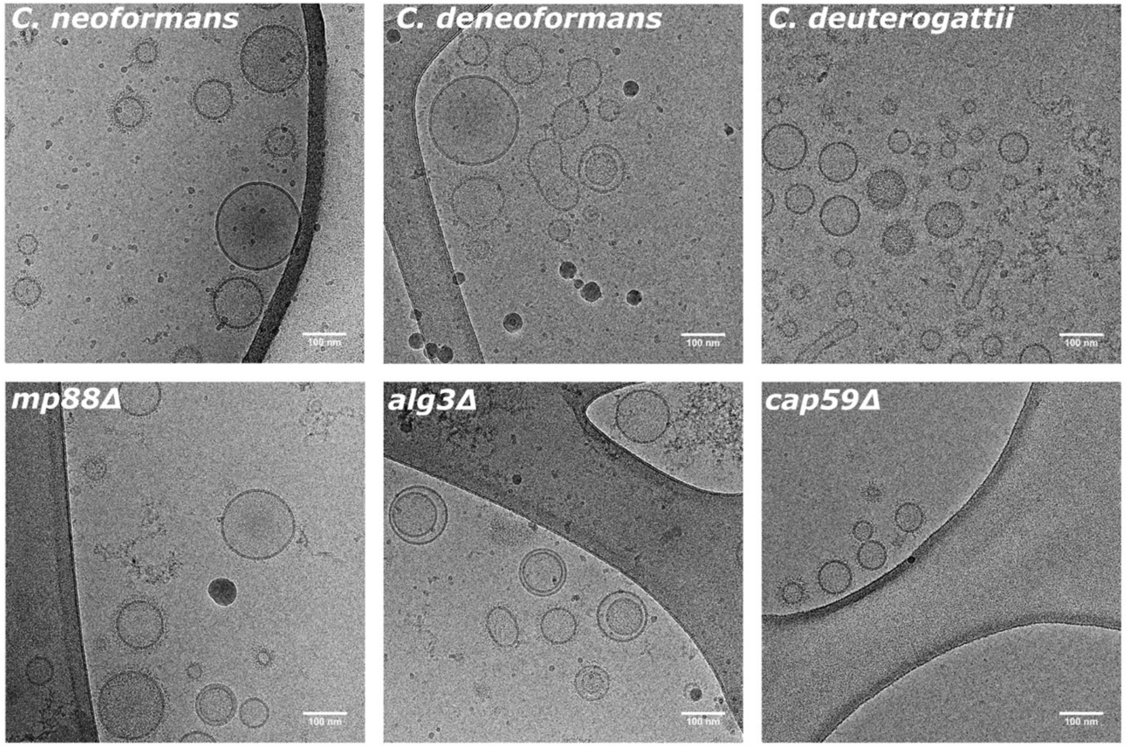

Figure S2. Representative cryo-EM images of EVs purified from different *Cryptococcus* strains.
Scale bars represent 100 nm. The data presented in this figure have been generated using images obtained using a TECNAI F20 transmission electron microscope
