# Supplementary material for: Cryptococcus extracellular vesicles properties and their use as vaccine platforms
Source: J Extracell Vesicles. 2021 Aug 2;10(10):e12129. doi: 10.1002/jev2.12129 (PMC8329992; doi:10.1002/jev2.12129)
Supplement: Supplementary file 3 — Supporting Information [file JEV2-10-e12129-s004.docx]

Table S3 (Rizzo et al)

Primers used in this study

CNAG_00776_Ex

agcgaggcactataaaccaggagag

CNAG_00776_5-5

GTAACGCCAGGGTTTTCCCAGTCACGACGataaggcaaatgggcagagggaagg

CNAG_00776_5-3

**ATCCACTTAACGTTACTGAAATCTCCTTC**tatgaataggttgtctagaag

CNAG_00776_3-5

**ATTTTTGAACAAACTCGAGTTACC**atgatctccaaggttgctgtcggcg

CNAG_00776_MKRr

cgccgacagcaaccttggagatca**GGTAACTCGAGTTTGTTCAAAAAT**

CNAG_00776_3-3

GCGGATAACAATTTCACACAGGAAACAGCgcagaatcaccgtcacactcagtg

CNAG_00776_ ex2

ggtaaacgtaccagtgtatcgctgc

gCNAG_00776-1

CATACACCGGCAGGGTATACTGTTGgctgctgccgccctcatggcGTTTTAGAGCTAGAAATAGCAAGTT

gCNAG_00776-2

CATACACCGGCAGGGTATACTGTTGagctgccggaagaggaagagGTTTTAGAGCTAGAAATAGCAAGTT
