# Supplementary material for: Cryptococcus extracellular vesicles properties and their use as vaccine platforms
Source: J Extracell Vesicles. 2021 Aug 2;10(10):e12129. doi: 10.1002/jev2.12129 (PMC8329992; doi:10.1002/jev2.12129)
Supplement: Supplementary file 4 — Supporting Information [file JEV2-10-e12129-s003.docx]

Table S2 (Rizzo et al).

*Cryptococcus* EV diversity

| **EV morphological aspects (%)** | | | | | | | |
| --- | --- | --- | --- | --- | --- | --- | --- |
| Strain | Total Regular | Total Irregular | Short Tubular | Long tubular | Flat | Multilayer | Miscellaneous |
| *C. neo* | 89.0 | 11.0 | 3.7 | 2.1 | 2.6 | 2.6 | 0.0 |
| *C. deneo* | 84.6 | 15.4 | 2.4 | 2.4 | 0.8 | 8.1 | 1.6 |
| *C. deutero* | 86.2 | 13.8 | 2.1 | 3.4 | 2.1 | 6.2 | 0.0 |
| *mp88∆* | 71.5 | 28.5 | 10.3 | 3.0 | 0.6 | 12.1 | 2.4 |
| *alg3∆* | 79.9 | 20.1 | 3.5 | 2.8 | 0.7 | 12.5 | 0.7 |

| *EV decoration aspects (%)* | | |
| --- | --- | --- |
| Strain | Decorated | Non-decorated |
| *C. neo* | 84.3 | 15.7 |
| *C. deneo* | 72.4 | 27.6 |
| *C. deutero* | 81.4 | 18.6 |
| *mp88∆* | 72.7 | 27.3 |
| *alg3∆* | 75.7 | 24.3 |
